# Exploring Parental Perceptions and Concerns About Sexuality and Reproductive Health of Their Child With Intellectual and Developmental Disability (IDD) in Mumbai

**DOI:** 10.3389/fsoc.2019.00058

**Published:** 2019-08-20

**Authors:** Pooja Menon, Muthusamy Sivakami

**Affiliations:** ^1^Sexuality and Disability, Rising Flame, Mumbai, India; ^2^Centre for Health and Social Sciences, School of Health Systems Studies, Tata Institute of Social Sciences, Mumbai, India

**Keywords:** sexuality, intellectual and developmental disabilities, reproductive health, parental perception, health rights

## Abstract

In India, sexuality is defined by society, which considers it as a taboo and is usually restricted to sex and related issues like sexual abuse, safe sex, unwanted pregnancy, etc. For a person with disability, sexual desires and wish for parenthood are considered as uncommon. Persons with intellectual and developmental disability (IDD) are characterized by subnormal intelligence, which may partially or totally restrict the person's ability to perform day-to-day activities and take life decisions. Thus, perceptions of primary caregivers, who take decisions on behalf of the person with IDD, have an important role in their life. The aim of the study is to understand parents' perceptions and concerns about the sexuality and reproductive health of their child with an IDD. The study adopted a qualitative methodology wherein 14 primary caregivers of individuals with IDD belonging to different socio-economic and demographic backgrounds were interviewed in Mumbai. The common perceptions were that puberty is expected, sexual behavior is unexpected, and there is a hope for cure. The reaction to puberty onset appeared to vary with the gender of the child. Puberty onset was often seen as an enabling factor for marriage especially among parents of female child. Marriage and/or childbirth was perceived as a possible cure for IDD by some parents. The dominant parental concerns were found to be safety, early onset of puberty, perception of child's action by others, and concerns about the child's family life. These concerns were also found to vary with the gender of the child, type of disability, and the socio-economic status of the family. Some of the perceptions about sexuality were shaped by the primary caregivers' concern for the individual with IDD.

## Introduction

As per the Rights of Persons with Disability Act, India (2016), intellectual disability is defined as a condition characterized by significant limitation in both intellectual functioning (reasoning, learning, problem solving) and adaptive behavior, which covers a range of everyday social and practical skills. It includes specific learning disabilities, i.e., conditions wherein there is a deficit in processing language, spoken or written, that may manifest itself as a difficulty to comprehend, speak, read, write, spell, or do mathematical calculations. It also includes “autism spectrum disorder,” a neuro-developmental condition that significantly affects a person's ability to communicate, understand relationships, and relate to others, and is frequently associated with unusual or stereotypical rituals or behaviors (Rights of Persons with Disability Act, India, 2016). As per The Arc, intellectual disability is also referred to as developmental disability, which is a broader term that includes autism spectrum disorders (ASD), epilepsy, cerebral palsy, developmental delay, fetal alcohol syndrome (or FASD), and other disorders that occur during the developmental period (birth to age 18). The major differences are in the age of onset, the severity of limitations, and the fact that a person with a developmental disability definition may or may not have a low intelligence quotient (I.Q.) (The Arc, 2018). For the purpose of this study, the condition would be collectively termed as “Intellectual and Developmental Disabilities” (IDD) (American Association on Intellectual and Developmental Disabilities, 2012). The National Sample Survey Organization (NSSO) estimates that, currently, 1.8% of the Indian population is disabled, yet the data may not be completely accurate as those with a mild degree of disability remain unidentified. A wide variation in prevalence of intellectual disability has been observed (1/1,000 to 32/1,000) depending on the case definition, methodology, and population selected (Girimaji and Srinath, 2010).

Considering a huge population of 1.3 billion, the difference of 1–2 % in prevalence is very large with the potential to significantly affect government policies and services for this population in India. According to 2011 census, total cases with IDD^1^ in India was found to be 1,505,964 (870,898 males and 635,066 females). Rural, underprivileged areas and males account for maximum cases of intellectual disability. As per definitions in the census data, cases of autism as well as cases where the respondent was unable to report the exact nature of disability were counted under “Any Other” section, which amounted to 4,927,589 (Office of the Registrar General and Census Commissioner, 2011). It can thus be safely assumed that cases of IDD reported are less than the actual number of cases. The discrepancy can be attributed to discordant definitions of disability, lack of awareness, and stigma associated with being identified as someone with an intellectual disability. This leads to IDD not being acknowledged either due to ambiguity about the condition or deliberate misrepresentation, which results in underreporting in India (World Health Organisation, 2011).

### Disability and Stigma

In India, there is a very strong belief in the metaphysical causation. The theory of karma is one such belief that is invoked to explain occurrence of disability. People accept disability as something that has resulted from their past karma. This belief in turn affects their motivation to overcome and/or deal with their disability (Berry and Dalal, 1996).

Perception of stigma and the experience of disability may vary with social determinants of the caregiver and person with disability. Parents of children with IDD in Mysore, India, reportedly faced a great deal of stigma, financial hardships, and social isolation. Factors that positively impacted the parents' ability to cope included higher education, religious faith, and social support (Venkatesh, 2008). Fear of stigma often manifests in the form of self-imposed isolation from the society as well as denial of intellectual disability in the child. Caregivers in Vellore, Tamil Nadu referred to intellectual disabilities as poor mental development, impaired brain development, slow learner, and lower intelligence. They felt that terming or accepting the condition as “Intellectual Disability” or “Mental Disability” or “Mental Retardation” would create stigma (Edwardraj et al., 2010). Another study found that parents of children with intellectual disability in Tamil Nadu have a dual burden—to protect and take care of their dependent child and also to guard their child against the negative attitudes of society that stem from lack of information and stigma (Suresh et al., 2017). This reinforces the idea that stigma associated with disability contributes to the underrepresentation of IDD in census while also contributing to social isolation of an individual with IDD.

### Sexuality and Reproductive Health Perceptions

According to World Health Organization (2006), sexual health is a state of physical, emotional, mental, and social well-being in relation to sexuality; it is not merely the absence of disease, dysfunction, or infirmity. Reproductive health is a state of complete physical, mental, and social well-being and not merely the absence of disease or infirmity, in all matters relating to the reproductive system and to its functions and processes. Reproductive health implies that people can have a satisfying and safe sex life and that they have the capability to reproduce and the freedom to decide if, when, and how often to do so (World Health Organization, 2006). Though parents have the primary responsibility of imparting sex education to their children, most young people in India derive their information about sex and sex behavior largely from companions, street-corner conversation, movies, and magazines (The International Encyclopaedia of Sexuality; 1997–2001)^2^. Adolescents with intellectual disability are often isolated from others of their age, which could lead to lack of opportunities to learn about their sexuality or to engage in social activities or sexual experimentation (Woodard, 2010).

Historically, persons with disabilities have been regarded by society in two contradictory ways about sexuality—either as asexual or as sexually threatening via cultural representations. They are also simultaneously seen as evil and innocent via different cultural lenses based on them being connected to the fruit of wrongdoings in the past life or as a god-sent for the fulfillment of a specific purpose. These views coupled with lack of realization of the individual's sexuality put them at a higher risk of sexual abuse (Rottenberg, 2012). Sexual autonomy is rarely conferred upon persons with disability, particularly mental disability, and reproductive choices are considered outside the purview of their mental abilities. The paradoxical views about sexuality of people with mental disability often leads to questioning the importance of sex education for them. As a result, children with intellectual or developmental disability are often excluded from sex education at home and at schools. Risk of sexual abuse, however, is real and is recognized by caretakers/family, which often leads to decisions like hysterectomy or abortions being taken without the consent of the person with mental disability (Kothari, 2014).

Parents of children with intellectual or developmental disability begin talking about sexuality only when their children begin to exhibit socially unacceptable behavior like indecent exposure, inappropriate touching, etc. Main areas of concern for parents are abuse, menstruation management, and socially unacceptable behavior (Talking about Reproductive and Sexual Health Issues, 2010). Often, parents of children with IDD do not recognize acts like kissing and hugging strangers impulsively and proposing marriage to strangers or imaginary people as being of sexual or romantic nature. Such acts are often written off as playing (Foundation for Social Transformation, 2015-2016). Parents of children with intellectual disability in Turkey revealed that 32% of the study population did not discuss sexuality with their intellectually disabled child. All the parents were concerned about the child's safety, but none were trained with respect to administering sex education (Aysegul et al., 2009).

### Gender, Disability, and Sexuality

In a patriarchal setting like India, women have lower status; disability makes them highly vulnerable as they are dependent on others. Males with disability are at relative advantage in terms of marriage, family formation, access to health, etc. as compared to females. Often, women with disability are not encouraged to form relationships, and it is considered that the only way a sexual relationship is sanctioned is through marriage (Youth Ki Awaaz, 2019). In a traditional Indian society, a series of ceremonies and rites initiate the adolescents into their sexual roles. These include instruction on marriage customs, sexual morality, and acceptable sexual behavior. Masturbation, while generally accepted and considered as preparation for mature sex life for men, is unacceptable among girls. In terms of marriage, it is expected that a husband must be able to earn a living and his wife must be able to run the home, which they set up after marriage (The International Encyclopaedia of Sexuality; 1997–2001)^2^.

Apart from paradoxical views about the existence of disability and sexuality of individuals with IDD, there also exist several misconceptions surrounding cure of disability in relation with marriage and sexual activity. Some Asian parents in the United Kingdom believed that developmental disability was curable, and marriage was one such remedy (Beber and Biswa, 2009). According to 2011 Census, only 47% of the disabled were currently married, whereas a huge percentage, i.e., 42%, were never married and 10% were widowed. The data also show that the percentage of disabled male getting married (62%) are significantly higher than disabled females (54%) (Office of the Registrar General and Census Commissioner, 2011).

### Sexual and Reproductive Health, and Safety Concerns

Studies point out instances of individuals with intellectual disabilities perpetrating or suspected to have perpetuated sexual crimes against people with or without disabilities (Furey and Niesen, 1994). An Australian study found that to mitigate the risk of sexual abuse, parents often resorted to overprotection and segregation of their daughter with IDD. It was, however, found that practices like overprotection, segregation, and training to be compliant with authority at home or at work, and the paradoxical view that they are sexual and asexual simultaneously increased the risk of abuse rather than prevent it (Chenoweth, 1996). A study in Australia reveals that the prevalent perception is that females with intellectual disability require more care in terms of healthcare and safety, while in case of males, their issues were studied as something that cause trouble to the society, thus problematizing their behavior. The male-oriented articles spoke extensively about instances of masturbation in public and masturbation to the point of injury (Wilson et al., 2010).

Type of disability and the cognitive traits accompanying it is one of the major factors that not only determine the consequences experienced due to disability but also shape parents' concerns and perceptions. A 2013 study in Orissa, consisting of 595 physically disabled and 134 intellectually disabled women, found that women with intellectual disability were at a higher risk of sexual abuse. Among physically disabled women, 13% reported rape, compared to 25% among the intellectually disabled women, even though the proportion of the intellectually disabled women was significantly lower in the study (Mohapatra and Mohanty, 2013). A Bangalore study found that reporting of sexual abuse in cases of a victim with IDD was not because the victim identified the incident as being wrong, but because it caused physical pain or discomfort. In the absence of discomfort, the incident would've gone unreported. In this case, the disability influenced the ability of the victim to identify the incident as abuse or wrong. However, there is a possibility of misreporting or lack of reporting of the crime, which may culminate in unwanted pregnancy or repeat abuse (Madar, 2017). A review study in India found that different disability types had different dynamics of abuse in operation. Physically disabled women may be limited by their inability to physically escape violent situations, while the visually impaired might not be able to correctly identify the perpetrator, or the hearing impaired who are also often speech impaired, who might not be able to shout for help in situations of abuse. These factors could have a bearing on ability to defend oneself or report the crime (Rao, 2004). Type of disability could thus increase the risk of abuse by influencing the ability of the victim to perceive the abuse, and report the abuse and hence the parental concerns regarding safety or their child may vary with the type of disability.

It is thus evident from literature review that the sexual and reproductive health concerns for individuals with IDD were either from a protectionist point of view or from a problematizing point of view. The nature of disability or the type of disability may influence parental concerns as it may impact the way the individual with IDD themselves perceives matters of sexuality, reproduction, and safety.

### Research Gap and Objective

Societal norms, stereotypes, and gender roles often govern perceptions about sexuality of people with disabilities. The sexual rights of those with IDD are even less acknowledged. This affects their ability to live a full healthy life. It is also apparent that disabled individuals are at risk of sexual abuse and can commit sexual offenses. They are thus vulnerable to sexually transmitted infections and unwanted pregnancies.

In India, IDD remains unexplored by studies and surveys conducted by the government. However, reliable estimates are warranted given the rate at which cases are diagnosed (Parva, 2017). While other disabilities like sensory and locomotor disabilities have been studied to a certain extent in India, the population with IDD has been excluded from most studies. This can be attributed to the stigma surrounding disability, underrepresentation of the segment in the general population, and taboo around sexual and reproductive health. It is also apparent that disabled individuals are at risk of sexual abuse and can commit sexual offenses. They are thus vulnerable to sexually transmitted infections and unwanted pregnancies. There is shortage of personal accounts as well as caregiver accounts about sexuality and reproductive health perceptions and concerns of people with intellectual or developmental disability in India. Sexuality and Disability in the Indian Context 2018 states that there is a dearth of voices of people with intellectual disabilities in the sexuality space. Studies where the participants are persons with disabilities often exclude people with intellectual or developmental disabilities due to ethical considerations (Talking about Reproductive and Sexual Health Issues, 2018).

Keeping this in mind, the objective of the present paper is to explore prime parental perceptions and concerns about sexuality and reproductive health of their child with IDD. For the purpose of this study, perception represents the way of understanding an event in the life of the person with IDD. A concern represents a feeling of worry regarding a life event in the life of the person with IDD.

## Methods

### Study Design and Setting

In Maharashtra, the highest number of people with IDD was in Pune with 11,128 (6,284 males and 4,844 females) followed by Mumbai with 10,460 persons (6,200 males and 4,260 females) (Registrar Governor of India, 2011). A study of this nature has not been conducted in either of the cities. The diverse population coupled with the fact that Mumbai has the second highest number of cases of intellectual disability made it an ideal location for the study. The study adopted an inductive approach using qualitative methodology.

### Participants and Participant Recruitment

The primary caregivers, i.e., parents/guardians of individuals with IDD, were the participants of the study. Participants were approached using purposive sampling via organizations and schools working with individuals with an IDD in Mumbai in April–May 2017. Those agreeing to help with the study were requested to provide details of parents who may be interested in participating. The schools and organizations shared the contact details of prospective participants after seeking consent from participants, after which the primary caregivers were approached for the study. The primary caregiver was asked to participate in the study after explaining the study in detail. The criteria were that the child of the participant must be diagnosed with an IDD. The diagnosis was confirmed by cross-checking with the organization referring the participants.

### Sample Size and Sample Characteristics

A total of 14 primary caregivers agreed to participate in the study. The participants belonged to diverse socio-economic backgrounds and played the role of caretakers to a child with IDD. The participants were disaggregated based on socio-economic status based on employment sector, place of residence, and education. Characteristics of the child with IDD were disaggregated based on age of the child, number of siblings, and disability as reported by the parents and as reported by the NGO/school. No disability certificate was reviewed at the time of recording the disability of the child.

Among the participants interviewed, nine were mothers, one was a guardian, and two sets were parents to an individual with IDD (see Table 1). Out of the 14 participants, 7 were employed in the formal sector while 7 were employed in the informal sector as vegetable vendors, laborers, daily wage workers, etc. Only seven participants had passed the 10th grade out of the 14. Out of the 14 participants, 8 were caregivers to a female child while 5 were caregivers to a male child. One participant had two daughters with an IDD. One child was below 10 years of age while there were seven and five individuals in the 10–20 and 21–30 age group, respectively. The disabilities covered in the participant universe were Down syndrome, autism, and a combination of disabilities like autism, IDD, mutism, etc.

**Table 1:** Parent and child socio-economic and demographic characteristics.

**PRIMARY CAREGIVERS CHARACHTERISTICS (*****N*** **=** **14)**	
Only Mothers	9
Mother + Father	4 (2 sets of parents)
Guardian	1
**Total**	14
**SOCIO-ECONOMIC STATUS**	
**Residence**	
Flat/Apartment	7
Slum	3
Chawl	4
**Education**	
Below 10th grade	7
Above 10th grade	5
**Employment**	
Parent employed in the formal sector (service/business)	7
Parent employed in the informal sector (daily wage workers/rag pickers/vegetable vendor, etc.).	7
**Religion**	
Hindu	6
Muslim	4
Catholic	4
**Socio-economic status(based on employment sector)**	
Low socio-economic status	6
High socio-economic status	8
**CHILDREN WITH IDD (*****N*** **=** **13)^a^**	
**Sex**	
Male	5
Female	8
**Age**	
Below 10 years	1
10–20 years	7
21–30 years	5
**Siblings**	
No sibling	5
One sibling	8
**Disability (as described by participant)**	
Autism	5
Delayed milestones	2
IDD^b^	4
Down Syndrome	1
Multiple Disabilities^c^	2
**Disability (as described by NGO/school recommending the participants)**	
Autism	4
Autism + IDD	2
IDD + Mutism^d^	5
Down Syndrome	2

a *One participant had two children with IDD*.

b *Term used originally was “Mental Retardation”. Given the regressive tone, it has been replaced with “IDD” in Table 1*.

c *Refers to circumstances where the child has additional disabilities like inability to speak or walk properly, etc., along with the IDD*.

d *Mutism referred to a condition where the person with IDD would not speak despite not having a speech impairment*.

### Ethical Considerations and Interview Procedure

Written informed consent form was obtained from the participants before commencing the interviews. The identity of the participants was not revealed at any point in the study. The children of the participants were not interviewed at any point in the study. Names of the schools and organizations through which participants were contacted were also not disclosed.

In-depth interviews were conducted with the participants. In cases where both parents were reported as primary caregivers, both were interviewed together. The interviews were audio recorded whenever the participants permitted. In the absence of permission to audio-record, the first author made notes during the interviews and detailed transcript of the interview was prepared immediately after the interview was conducted. In case where the interviews were conducted in Hindi, the transcripts were translated into English prior to analysis.

### Interview Schedule

The interview schedule is composed of a section that gathered demographic and socio-economic information about the participant and their child such as age, nature of disability, source of income, etc. The next section explored the thoughts and concerns the participants had on sexuality of the child by probing into their thoughts and reaction to puberty onset, their concerns regarding social interactions post puberty, etc. The final section explored the thoughts and concerns regarding marriage, intimate relations, and reproduction by probing into the feasibility of marriage and childbirth for the child as well as interest expressed in the same by the child with IDD.

### Data Processing and Analysis

Qualitative data analysis was done using thematic analysis. The transcripts were thoroughly examined and openly coded based on the emerging ideas. The codes were developed in consultations with the second author, and similar codes were merged. Larger themes and sub-themes were then identified by the authors. These categories and themes were further examined to ensure their alignment with the objectives of the study. The in-depth interviews were analyzed using Atlas TI version 9 and MS Excel.

## Results

**Figure 1:**
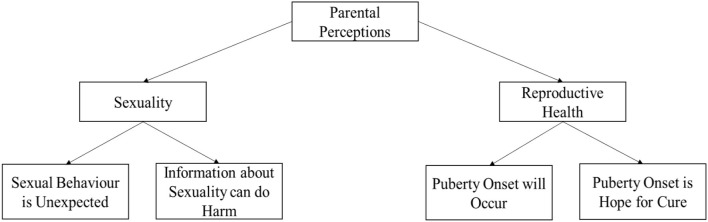
Parental perceptions.

**Figure 2:**
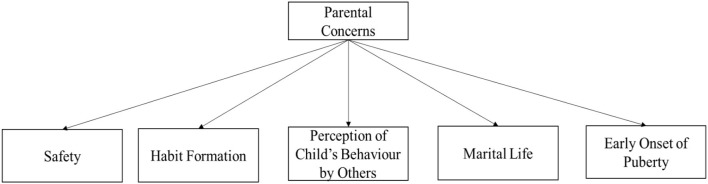
Parental concerns.

The study identified four major themes (Figure 1) regarding parental perceptions about sexuality and reproductive health of their children with IDD. Parental concerns were found to revolve around five themes (Figure 2). These themes were found to vary with the gender of the child, type of disability, and socio-economic status of the family. Perceptions and concerns appeared to be the same across age group, religions, and family structures of the participants. Educational qualification of the primary caregiver was not an influencer about sexuality and reproductive health perceptions and concerns. This could be attributed to the fact that sex education as a formal subject has only been introduced recently in educational systems.

### Parental Perceptions About Sexuality and Reproductive Health

#### Puberty Onset Will Occur

Puberty was expected by all the parents. The response to onset of puberty however varied with gender of the child. When it came to a female child, puberty onset was celebrated often, with parents expressing relief about the fact that the girl will be able to bare children and thus it is possible to get the girl married. This can be demonstrated as follows:

“I knew it (menstruation onset) would happen, I was happy that everything else was fine with her body. She cannot understand things but everything else is working fine.” (Mother—Female Child with Autism—High Socio-Economic Status)

“I was relieved, now it is possible to get her married, everything works fine, and she looks also normal.” (Mother—Female Child with Autism—Low Socio-Economic Status)

When it came to a male child, the reaction to onset was worries about mood swings and rebellion. Coupled with increasing physical strength, parents expressed first reaction being that of worry about how to manage the child in the event of an emotional outburst.

“He came to me one day saying he had hair in his armpit. I told him it's normal, that he is a growing boy.” (Mother—Male Child with Autism—Low Socio-Economic Status)

*“I knew he would get puberty. But when he started changing my first thought was behavioral issues. How will we manage if he refuses to obey or gets angry, he is getting stronger you see?” (Father—Male Child with IDD*+*Mutism—High Socio-Economic Status)*

#### Sexual Behavior Is Unexpected

Sexual behavior includes self-stimulation, peeping while members of opposite sex are undressing or bathing, viewing porn, and expression of desire to have sex. Parents of boys stated instances where they observed nightfalls and morning erections. Parents of all except one boy reported that while they expected hair growth and voice changing, they did not expect nightfalls or erections. Parents often did not know how to react to the questions posed by the child on occurrence of nightfalls or erections. Affinity toward intimacy was also something the parents did not expect. This can be demonstrated as follows:

“Few times when I go to wake him up, I notice that he was (whispers) hard. Once he asked his dad why it (penis) was standing the way it was, he didn't know how to explain it to him. I was not expecting this. I don't want to teach him too much about it also, being a boy, he will keep doing this all day then.”(Mother—Male Child Autism—High Socio-Economic Status)

“There was this one time I saw him look into the neighboring house. The couple was being intimate, and he saw that. I scolded him a lot that day. But after that I have noticed him kiss the pictures like that, like he saw there. I did not expect this.” (Father—Male Child with Down Syndrome—Low Socio-Economic Status)

*“All (arousal, stimulation, sexual needs) such thoughts rarely cross normal girl's minds, she is not normal. I don't think she is interested in all this (sex, arousal), basic things also she doesn't know or understand, all this is beyond her.” (Mother—Female Child with IDD*+*Mutism—High Socio-Economic Status)*

Parents of a female child do not expect sexual behavior/sexual interest of any kind from their child. Actions or incidents of sexual nature were often written off as playfulness or inappropriate. This can be demonstrated as follows:

*“There was an instance where the teacher said she was rubbing herself against other children. She is a friendly girl you see. I asked her to not do that. Others may not see it as being playful.” (Mother—Female Child with IDD*+*Mutism—High Socio-Economic Status)*

“She doesn't express anything about all this (when asked about desire for intimacy and relationships).I hope she gets a good and patient man. She does as she is asked. She will get used to it (sexual intimacy) once she gets married.” (Mother—Female Child with Autism—Low Socio-Economic Status)

#### Puberty Onset Is Hope for Cure

There was a notion that marriage and/or childbirth can cure IDD. This was also a reason why onset of puberty in girls was celebrated as the parents felt it increased their chances of getting her married. It was believed that taking up responsibility in the form of a spouse or a child would help cure IDD. This can be demonstrated as follows:

*“Maybe he will get cured after marriage. I have heard of cases where children are cured after marriage or after having relations (sexual relations) and children.” (Mother—Male Child with Autism*+*IDD—Low Socio-Economic Status)*

*“She has matured now. She is a pretty girl and obedient, never back answers. We hope to be able to find a good man for her. Maybe she will get cured after marriage. Her in-laws will be happy with her.” (Mother—Female Child with IDD*+*Mutism—Low Socio-Economic Status)*

*“Maybe we find someone for him, I have heard that having relationships can cure such illness like he has. If we pay someone to come over and provide that relationship, then maybe he can be cured.” (Father—Male Child with IDD*+*Mutism—Low Socio-Economic Status)*

*“Miracles happen, we can always be hopeful. I have heard that in kids like her, once you take responsibilities (get married/have children) the illness goes away. I hope that happens someday” (Mother—Female Child with IDD*+*Mutism—High Socio-Economic Status)*

#### Information About Sexuality Can Do Harm

Participants believed that information about sexuality was not as important as information about safety or/and socially acceptable public behavior. They felt that topics about sexuality like masturbation, the physical act of sex, and how a child is conceived were irrelevant to their child as he/she may not be able to comprehend it or he/she may never get married. In some cases, because the parents themselves were never given sex education, they believed that sex education was unnecessary. Parents feared that giving information about sex before marriage could lead to experimentation or increase the risk of abuse.

*“Better to teach more about how to behave in public so that she can be safe. This information about sex and all this is not going to help her. She won't be able to understand anyways.” (Mother—Female Child with IDD*+*Mutism—Low Socio-Economic Status)*

*“He tends to repeat things. If we teach him about sex or stimulation, he will keep doing it again and again. He doesn't know when to stop things.” (Mother—Male Child with Autism*+*IDD—High Socio-Economic Status)*

“All this (information about sex) need not be taught, we were never taught. We knew after marriage what needs to be done. Her husband will know should we plan to get her married. He will teach her. If I am not able to get her married, I won't tell her anything at all, I wouldn't want her to try it (sex) out of curiosity.” (Mother—Female Child with Autism—Low Socio-Economic Status)

### Parental Concerns

#### Safety

Parents of both boys and girls feared abuse. Although abuse awareness was taught in school for children, parents doubted the ability of their child to grasp and remember what was being taught. Parents also feared that the child may not report instances of sexual abuse as they may not be able to identify it as abuse or they may not know how to communicate the incidence to authorities. Hence, parents often resorted to never leaving the child alone for as long as possible as a way of protecting them. This can be demonstrated as follows:

**“***My daughter does not speak. If something happens, she won't even be able to call for help let alone give a statement in the aftermath of the event. All this is given that she recognizes that what is happening is wrong.” (Mother—Female Child with IDD*+*Mutism—High Socio-Economic Status)*

*“Of course, we fear sexual abuse, but he is being taught good touch bad touch, and he is obedient and rarely goes out without us, so I am thinking we can hopefully manage that.” (Mother—Male Child with Autism*+*IDD—Low Socio-Economic Status)*

*“She has an intellectual problem, what if she does not understand what happened? She could become pregnant. Who will believe her given her mental condition? What will we do then?” (Mother—Female Child with IDD*+*Mutism—Low Socio-Economic Status)*

#### Habit Formation

Parents of male children feared habit formation when it came to actions like self-stimulation. They thus felt that sex education would be harmful for them. They felt that the child would self-stimulate in public or seek someone to fulfill their sexual urges, which would lead to the child getting into trouble with the law and with the society. Such concerns, however, were not raised by parents of female child as the perception was that a girl will not display sexual behavior publicly. This can be demonstrated as follows:

*“A friend told me she knew someone whose son was like my son, they taught him about sex and then he started seeking sex from everyone. He wanted to try. Finally, his mother had to provide herself to him to fulfill his urges. I cannot do things like this.” (Mother—Male Child with IDD*+*Mutism—High Socio-Economic Status)*

“She is a girl, girls don't feel things like boys, she won't express all this (sexual desires) publicly, there is no need to teach her unless we plan to get her married, which as of now we can't afford.” (Mother—Female Child with Autism—Low Socio-Economic Status)

#### Perception of Child's Actions by Others

Parents of boys feared that once the child starts appearing like a man, his actions could be misconceived as lecherous or perverse when that may not be the case. Many of the boys played with children who matched their mental age and not their chronological age. This was a major source of concern for many parents. This can be demonstrated as follows:

*“People will not see that he doesn't understand not to do it (self-stimulate) in public. Today we are there to explain to others that he has a problem, but after us if this continues, who will save him. What if he wants to do it with someone else and not just self-stimulate? How I will find someone unless we get him married.” (Father—Male Child with Autism*+*IDD—Low Socio-Economic Status)*

“If you see him, you won't feel there is anything wrong with him. People won't know that he does not know what he is doing. The other day he lifted the skirt of the neighbor's maid, they thought he had bad intentions, but he was just trying to search for his ball. People don't see that because he looks normal.” (Mother—Male Child with Autism—High Socio-Economic Status)

#### Marital Life

Concerns about the life of the child with spouse and in-laws after marriage and/or childbirth were a common problem for parents who wanted to get their children married. The concerns about marital life varied based on gender of the child and the socio-economic status of the family. While parents of female children were worried about adjustment and acceptance into the new family, the parents of many male children were concerned about finding a spouse who was normal and/or beautiful and preferably from a low socio-economic status. Families with a low socio-economic status worried about dowry and hoped to compensate for their daughter's disability with qualities like keeping the in-laws happy, housekeeping, and childbearing. Appearance, presence of disability, and financial stability did not appear to be a matter of concern for parents of female children. Higher socio-economic status families feared that they would get proposals from money-diggers and it would be difficult to sort out the genuine proposals. It was not uncommon for them to expect non-disabled spouses from low socio-economic status family for their child as they were ready to offer complete financial support.

This can be demonstrated as follows:

“She looks different that is all. She can take care of herself but I hope she finds someone who goes beyond her looks. In that case we can get her married.” (Guardian—Female Child with Down Syndrome—High Socio-Economic Status)

*“If we find a normal girl then yes, we can get him married. We cannot handle two people like him. The girl will have all the facilities she could wish for here.” (Father—Male Child with Autism*+ *IDD—High Socio-Economic Status)*

*“We have enough money to be able to support grandchildren as well if needed, but we don't want someone to marry her only because of what she stands to inherit.” (Mother—Female Child with IDD*+*Mutism—High Socio-Economic Status)*

#### Early Onset of Puberty

Parents were concerned about the early onset of puberty as they feared that their child's body wouldn't be able to handle it. Such fear ranged to an extent that some of the participants were giving medicines to their daughters to suppress menses. This can be demonstrated as follows:

“No not yet (Puberty has not yet started). I am worried though, one of her classmates is only 9 and she had her period. I do not know what I would do if it starts so early for her.” (Mother—Female Child with Autism—Low Socio-Economic Status)

“She was 11 when her period started. I took her to the doctor immediately. He gave her injections to stop the period. She is too young to handle all this. We will stop the injections when she is older” (Mother—Female Child with Autism—High Socio-Economic Status)

## Strengths and Limitations

Themes about sexuality and reproductive health issues of individuals with IDD emerging from literature review were found to hold true in the Indian context where they are seldom explored. The study was also able to identify possible socio-demographic factors that impacted perceptions and concerns thus identifying future research avenues. Since studies exploring the intersection of sexuality and IDD in India are lacking, this study presents future research avenues. In doing so, this study adds values to the research area of disability and sexual and reproductive health. This study involves primary caregivers of children with IDD; however, the perceptions and views of the individual with IDD would have been very valuable to the study by bringing a direct disability perspective, though we could not interview them due to ethical issues.

Given the sensitive nature of the topic, it was extremely challenging to convince participants to be a part of the study, and when participants expressed interest to talk to the first author, rapport building to encourage discussion on sexual and reproductive health took an enormous amount of time. The absence of funding also restricted traveling to other cities to seek more participants. These reasons together restricted the sample size to only 14. However, we believe that the study was still able to bring out valuable findings in the field of sexual and reproductive health and IDD.

## Discussion

Adolescence is a challenging time in the life of any child. The physical changes coupled with the myriad of emotional changes make it difficult for the child to cope in the absence of proper guidance and care. When the child in question suffers from an IDD, adolescence poses a bigger challenge due to their dependence on others and lack of social skills. Such children require extensive care and guidance when it comes to puberty and sexuality, in the absence of which they may fail to attain full health. The access to guidance of this nature is subject to parental perceptions and concerns about these topics. This section discusses the results of this study as illustrated through Figures 1, 2.

### Puberty Is Expected While Sexuality Is Unexpected

It was observed that many parents consider sexuality and puberty to be two distinct unrelated things. While the onset of the latter is often celebrated, the expression of the former is frowned upon or discouraged after being received with initial shock and disbelief. Parents understand that puberty is something that will happen to their child, but they do not expect to see signs of sexuality like self-stimulation and arousal. Puberty is often awaited as it increases the chances of finding a spouse for their intellectually and/or developmentally disabled child. Information about puberty is thus readily given to the child either prior to or after the onset. Early onset of puberty, however, was a source of concern as parents believed that their daughter was too young to be able to deal with the physical discomfort and hormonal changes. Participants accepted using medicines to delay the onset of menses in the daughter with IDD.

Sexuality and sexual behavior are rarely expected as parents feel that their child is either asexual or not old enough mentally to be experiencing changes that are sexual in nature. Despite experiencing incidences of sexual display, the parents withhold information about sexuality and sexual behavior from the child. Parents often resort to denial where they do not perceive an act as being sexual and discourage the child from engaging in the activity. A common fear of parents as described by the providers was that if the child is taught self-stimulation, he/she may get addicted to it and engage in self-stimulation publicly. This agrees with existing literature that states that individuals with intellectual disabilities are often considered to be asexual, leading to them being excluded from sex education and reproductive health services (Albrecht, 2005; Rottenberg, 2012).

It is evident from this study that the link between sexual expression and puberty is less understood in the domain of IDD. Further studies in this domain are warranted to explore the reason why puberty is expected while sexual expression and sexual maturity were not expected.

### Safety Concerns Varied With the Gender of the Child

With respect to a male child with IDD, the dominant concern was that the child's actions could be misunderstood as hypersexuality or sexual misconduct. This appears to stem from the paradoxical view of hypersexuality coupled with prevalent gender norms that men are meant to be sexually expressive. The major concern was thus to protect their son from being misunderstood by others as a sexual offender due to uncontrolled expression of sexuality. Sexual abuse was also a concern but was not as dominant as in the case of a female child with IDD. Parents did not express the need to always keep their son under supervision.

With respect to a female child with IDD, the main concern was that the child must be kept safe. This often meant being kept in constant supervision. To ensure safety, some parents resorted to never leaving the child alone in the company of the opposite sex or never letting the child out of the house unaccompanied by a trusted family member. Parents believed that segregation from the opposite sex and constant supervision would help mitigate the risk of sexual abuse. This agrees with earlier literature that states that overprotection and segregation is a common practice with intellectually disabled female children (Chenoweth, 1996).

### Consent May Not Be Sought for Marriage and/or Childbirth

The topic of marriage and childbirth did not appear to be approached from a rights-based view. The decision to marry the child is made by the parents without necessarily taking the consent of the child or explaining the concept of marriage to the child.

Withholding sex education was a common practice stemming from personal experience of the participants never being formally taught about sexual health. Participants claimed to have figured it out via their social circles. Literature review states that adolescents with IDD have a lesser chance of learning about sexual health through social circles given their segregation and social isolation (Woodard, 2010). It is thus unlikely that an individual with IDD will be able to accumulate accurate and complete information about sexual and reproductive health in the absence of formal sex education. In the absence of information about sexual relations and childbirth, the individual with IDD may not understand the importance or the physical and emotional consequences of sexual intimacy for themselves. In the event of pregnancy, they may/may not be aware about the options they have with respect to continuing the pregnancy. A new baby brings with it a lot of responsibilities that take a toll physically and emotionally on the parents. A parent with IDD may or may not understand these requirements or the implications of the same on their life. This agrees with the findings of a study that found that women with disabilities were often denied their reproductive rights with cases of unintended pregnancy and forced abortions resulting from lack of sex education (Kothari, 2014; Talking about Reproductive and Sexual Health Issues, 2018).

### Socio-Economic Status Influenced Some Concerns and Perceptions

The parental concerns appeared to vary with the socio-economic status of the family. Participants belonging to the lower socio-economic status expressed that they hoped for a non-disabled spouse. The non-disabled spouse would be able to support the family financially and be able to take care of the parents in old age. Some participants felt that marriage was not feasible as they would not be able to support their child's family should the spouse also be disabled. For families residing in slums and chawls, communal washrooms, frequently changing residents, and poor security were a great source of safety concern for their child with IDD. Since hiring a caretaker or attendant was unaffordable, many mothers resorted to quitting their job in order to ensure that their child was always safe.

Participants with a high socio-economic status who wanted to get their child married were not concerned about being able to take care of their child after marriage. Some participants felt that their ability to secure the financial future of their child after marriage would help them find a non-disabled spouse for their child. Some participants planned to seek non-disabled spouses from the lower socio-economic status with the hope that financial security would make up for their child's IDD. Safety concerns were limited to not only sexual abuse but also financial exploitation at the hands of the spouse. The former, however, was addressed by hiring full-time caretakers, which was not the case for participants belonging to a lower socio-economic status.

A comparative study exploring how socio-economic status influences life decisions like marriage, parenthood, and education by parents of children with and without IDD can be fruitful in understanding the interplay between disability and socio-economic status.

### Marriage and Childbirth Can Cure IDD

A predominant belief reported among the parents and caregivers was that marriage could lead to the cure of IDD. The hope that childbirth or marriage can cure IDD appeared to stem from lack of awareness and prevalent beliefs that tie marriage and parenthood to the development of a sense of responsibility. This was the motive behind many parents' decision to get their child married. Childbirth was also something that is perceived to cure IDD. These findings agree with studies reporting marriage as a cure for IDD (Beber and Biswa, 2009; Foundation for Social Transformation, 2015-2016).

None of the parents who hoped for marriage raised concern about the child being born disabled. The implications of the birth of a disabled child on the parents with IDD were also not a source of concern. Birth of a disabled child could pose severe difficulties for the caretakers as well as the parents of the child. Lack of knowledge about childbirth as well as childcare could be strenuous both mentally and physically for a mother with IDD. There may also be a chance that if a non-disabled child is born, the child may be taken away from the parents with IDD because of their incapability to care for the child. Studies exploring such misconceptions and the impact of these misconceptions on lives of individuals with IDD in the Indian context are lacking and thus warrant further exploration.

### Interplay of Societal Norms and Sexuality

A woman is expected to conform to the societal standards of beauty while being complacent and obedient. She is expected to play the role of caregiver, wife, and mother. While a woman is expected to play the role of a wife and mother, she is not expected to be sexually expressive or have sexual desires. The family honor is often tied to the woman's adherence to these standards and her social conduct. A woman who is unable to adhere to these norms is often deemed as less suited for marriage. Any form of social misconduct may bring dishonor to the family. These norms appear to greatly influence parental perceptions and concerns about sexuality and reproductive health. These norms have been confirmed through literature review (The International Encyclopaedia of Sexuality; 1997–2001)^2^.

A woman with an IDD may or may not conform to these norms given the paradoxical views prevalent about her sexuality. The need to adhere to these standards may be the reason why many participants expressed relief that their daughter with IDD looked “normal.” Coupled with onset of puberty in the girl, it insinuated that the girl may conform to the role of a wife and mother as well. Some participants even inculcated values of compliance and obedience in their daughter to ensure that future in-laws and husband would have no complaints. The IDD, should it impact the physical features or behavioral attributes of the girl, was reported as a source of further concern as it would hamper marriage prospects. Self-stimulatory behavior like rubbing up against other people and objects were deemed inappropriate by parents but not seen as possible expressions of sexual desire. It was stated that the spouse would initiate intimacy and the woman in question would obey and get used to it eventually. Thus, sex education was considered as irrelevant especially for a female child with IDD. Consent and marital rape were also not a source of concern for participants wanting to get their daughter with IDD married.

For participants who did not wish to get their daughters married, sexual abuse and unwanted pregnancies were the major concern. Type of disability plays an important role when it comes to the ability of the child to report or escape sex abuse. In case of IDD or cases where IDD coexisted with conditions like mutism, the way the child would perceive sex abuse was a source of concern. The parents also voiced concerns about the ability of their child to report the abuse given their inability to perceive it as well as the accountability the child's account should he/she perceive it. This agrees with a study in Bangalore among individuals with sensory, locomotor, and intellectual disabilities, which stated that abuse was perceived differently by the victim based on their disability, which in turn influenced reporting of the crime to the family or the police (Madar, 2017). Participants feared that inability to report the incident could lead to repeat abuse or unwanted pregnancies. Hysterectomy was considered as a solution to this problem even though it would do nothing to prevent sexual abuse. While some participants raised concern regarding their child being able to report abuse, they did not understand the relevance of topics like consent and sex education for their daughter.

Traditionally, men are supposed to fulfill the roles of a provider to their family. Their masculinity is determined by their physical and emotional strength and sexual prowess, and they are expected to be defiant or rebellious. Expression of sexuality is natural in the case of males and is expected by participants by virtue of the child being a male but not expected by virtue of disability. Puberty onset was greeted with fear and concern that uncontrolled expression of sexuality could lead to isolation from the society or trouble with the law. Information about sex was withheld from male children as they felt that it could lead to experimentation especially in cases where the participants did not wish to get their son married. There were cases reported where a post-pubertal male child playing with children younger than him was not received well by the society as well as cases of the child trying to force himself on a family member. These incidents were attributed to exposure to sex in media. This agrees with the literature review that states that there are instances of individuals with intellectual disabilities perpetrating or suspected to have perpetuated sexual crimes against people with or without disabilities. This was also evident in a literature review that states that sexual behavior of males with disability is often perceived as being problematic whereas puberty in females is seen as warranting a protectionist and health centric approach (Furey and Niesen, 1994; Beber and Biswa, 2009).

How societal norms influence these perceptions in the absence of disability will better help identify societal determinants that govern sexuality. It is worth exploring if the same interplay of gender roles and sexuality is prevalent among parents with non-disabled children vs. children with IDD.

### Type of Disability May Influence Perceptions and Concerns

It was observed that the type and nature of disability may have a role to play in shaping concerns and perceptions. IDDs that had an impact on the physical appearance of the individual appeared to play a role when it came to marriage prospects for females and perception by others for males. This in turn influenced reaction to puberty onset. A female with IDD conforming to societal norms of beauty and social conduct was deemed as marriageable by parents as opposed to a female with IDD who did not conform to these standards. Behavioral traits of disability like panic attacks, repetitive behavior, emotional outbursts, etc. also shaped perceptions and concerns surrounding marriage and family life. Complacency and emotional stability contributed to social acceptability, thus increasing chances of marriage.

Disability type may impact how the victim of abuse reacts to abuse and thus influences parental concerns with respect to safety. The physical and behavioral traits associated with IDD may also influence outcome of such incidents for males and females. A person with IDD may or may not be able to describe the incident appropriately or identify the perpetrator. In case of multiple disabilities like mutism and IDD or physical disability and IDD, the individual may not be able to raise an alarm or defend themselves. If repetitive action is a trait of the IDD, the child may repeatedly indulge in self-stimulatory behavior if taught about the same. This coupled with the physical and behavioral traits of the male or female child could lead to their actions being perceived as intentional or inviting, respectively. Actions of an intellectually disabled male with no obvious physical or behavioral irregularity may have harsher repercussions compared to a male who has visible traits of IDD. This agrees with past studies in India that examined response to incidents of abuse, perception of the incident itself, and concerns for safety across different types of disability like sensory, locomotor, and intellectual disability. The studies found that reaction to incidents of abuse varied with type of disability among other things (Rao, 2004; Madar, 2017). This leads to parents feeling that sex education is harmful for their child. From a parental point, this increases risk of repeat abuse, unwanted pregnancy, and wrongful incarceration. Further studies are warranted to understand how these concerns and perceptions vary across disability type.

### Concerns Shaped Some Perceptions

It was observed that some parental perceptions were shaped based on their concerns. Parents feared that educating the child about masturbation could lead to habit formation, which could in turn result in the child's action being misunderstood by others. This can be attributed to the social taboos around expression of sexuality. Similarly, all parents feared for the safety of their child and that exposure to sexuality could lead to experimentation or forgetting of lessons on safety. These same reasons were cited as the justification for the perception that information about sex could be harmful as it could lead to wrongful incrimination or repeat abuse should it lead to habit formation or should the child not be able to perceive the act as abuse. Another concern was how the child would adjust to a new family after marriage. This concern appeared to influence the choice of activities that the child was being trained to carry out. There were cases where the girl was being taught to cook and clean, so she could please her in-laws, and where a boy was being taught to groom himself, so he could find a good-looking wife.

It may thus be possible to change or challenge perceptions by addressing the underlying concerns. Such concerns can be identified and addressed via research of the same in a population with IDD.

## Conclusion

**Figure 3:**
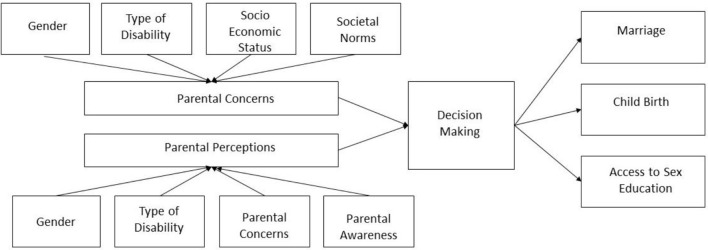
Factors affecting parental perceptions and concerns and its influence on decision making.

From the findings of this study, it can be concluded that parental perceptions about the sexuality and reproductive health of their child with IDD may influence the way important life decisions of their life are taken. The study was also able to identify factors that influence parental perceptions and concerns, thus highlighting future research. The interplay of the study findings are illustrated in Figure 3.

Factors like gender, type of disability, socio-economic status, awareness, and societal norms play a role when it comes to shaping parental perceptions and concerns about the sexuality and the reproductive health of their child with IDD. Lack of awareness about sexuality in the context of disability often leads to misconceptions like childbirth or marriage being a possible cure for IDD and individuals with IDD being asexual. Parental denial of sexual expressions by their child with IDD is common. Type of disability and gender influence both safety and marital life concerns. Depending on the type of disability, the individual may or may not be able to defend themselves or report abuse. For an individual with IDD, when the disability type impacts appearance, behavior, ability to earn a living, ability to take on the role of a caretaker, etc., it can impact marriage prospects.

These perceptions and concerns appear to influence life decisions like access to sex education, marriage, and childbirth. Lack of access to accurate sex education could lead to inability of the person with IDD to identify and report abuse, which could culminate in unwanted pregnancies. It could also limit the individual's ability to enjoy a healthy and satisfying marital life. Simultaneous denial of sex education and facilitation of marriage can result in the person with IDD being expected to fulfill roles they were not prepared for, leading to sexual abuse and/or unwanted pregnancies.

Research is warranted to understand the true impact these perceptions have on the sexual and reproductive health rights of people with IDD.

## Ethics Statement

The study has been approved by the Research Committee at the School of Health Systems Studies Tata Institute of Social Sciences for the first author's master's degree. Informed consent was obtained from all individual participants included in the study.

## Author Contributions

PM and MS conceived the study, designed the study and the data collection tools, and interpreted the data. PM collected and analyzed the data and wrote the first draft of the paper. MS critically revised subsequent drafts of the paper.

### Conflict of Interest Statement

The authors declare that the research was conducted in the absence of any commercial or financial relationships that could be construed as a potential conflict of interest.

## Footnotes

^1^Census 2011 has termed the disability as “Mental Retardation”. The authors recognize the word to be regressive. Thus, for the purpose of this study, the term “Mental Retardation” is replaced with the term “Intellectual and Developmental Disability (IDD)” when citing census data.

^2^Encyclopaedia of Sexuality. Available online at: http://www.sexarchive.info/IES/india.html#references (accessed October 12, 2017).
